# Histopathological validation of safe margin for nephron-sparing surgery based on individual tumor growth pattern

**DOI:** 10.1186/s12957-021-02375-3

**Published:** 2021-08-28

**Authors:** Gang Li, Tengfei Xiao, Keruo Wang, Renya Zhang, Aixiang Wang, Chengzhi Yan, Chunhui Wang

**Affiliations:** 1 Department of Urology, The Second Hospital of Tianjin Medical University, Tianjin Institute of Urology, Tianjin, 300211 China; 2 Department of Reproductive Health, W.F. Maternal and Child Health Hospital, Weifang, 261000 Shandong Province China; 3 Department of Pathology, The Affiliated Hospital of Jining Medical University, Jining, Shandong China; 4 Department of Pathology, The Second Hospital of Tianjin Medical University, Tianjin, China; 5 Tianjin Baodi Hospital of Tianjin Medical University, Tianjin, 301800 China; 6 Department of Urology, Affiliated Hospital of Chifeng University, Chifeng, China

**Keywords:** Renal cell carcinoma, Pathology, surgical, Nephrectomy, Surgical margin, Survival analysis

## Abstract

**Background:**

To evaluate the clinicopathologic value of morphological growth patterns of small renal cell carcinoma (sRCC) and determine the actual demand for taking a rim of healthy parenchyma to avoid positive SM.

**Methods:**

Data was collected from 560 sRCC patients who underwent laparoscopic surgeries from May 2010 to October 2017. One hundred forty-nine cases received nephron-sparing surgery (NSS) and others received radical nephrectomy (RN). All specimens were analyzed separately by two uropathologists, and three morphological growth patterns were identified. The presence of pseudocapsule (PC), surgical margins (SM), and other routine variables were recorded. The relationship between growth patterns and included variables was measured by the *χ*^2^ test and Fisher’s exact probability test. Survival outcomes were evaluated by Kaplan-Meier method and the log-rank test.

**Results:**

The median age of patients was 63.2 years old and the mean tumor diameter was 3.0 cm. Four hundred eighty (85.7%) cases were clear cell RCC and 541 (96.6%) cases were at the pT1a stage. Peritumoral PC was detected in 512 (92.5%) specimens, and the ratio of tumor invasion in PC in infiltration pattern increased obviously than that of the other growth patterns. Similarly, the pT stage was significantly correlated with the infiltration pattern as well. One hundred forty-nine patients underwent NSS and 3 (2.0%) of them showed positive SM after operation. Statistical differences of the 5-year overall survival (OS) and the cancer-specific survival (CSS) existed between different morphological growth patterns, PC status, and pT stages.

**Conclusions:**

Morphological growth patterns of sRCC might be used as a potential biomarker to help operate NSS to avoid the risk of positive SM. How to distinguish different morphological growth patterns before operation and the effectiveness of the growth pattern as a novel proposed parameter to direct NSS in sRCC patients deserves further exploration.

## Background

RCC is one of the most malignant tumors among genitourinary diseases, and significant progress in treatments has been achieved during the past 20 years. NSS has been widely confirmed as an effective measure for sRCC [1, 2], and the number of sRCC patients who accepted NSS has increased significantly in recent years. Nevertheless, no research had clarified the histopathological and clinical features of different kinds of morphological growth patterns of sRCC. For this regard, a more practicable classification and treatment strategy that could be utilized for choosing proper therapeutic methods for sRCC patients is necessary. A better understanding of the diverse growth patterns of sRCC, including their intricate characteristic, might bring about novel prognostic and therapeutic prospects.

Generally, the growth of tumor can be broadly divided into two groups according to the morphology of tumor and parenchyma relationship: the expansive growth pattern and the infiltrative pattern [3]. Relevant studies have shown that the layer of connective fibrous tissue termed tumor PC, located at the interface between the tumor and adjacent renal parenchyma [1, 4], and in some cases, the presence of tumor PC invasion, has been considered poor prognostic outcome for RCC [5, 6]. In the past few years, several studies have indicated a reduction of the thickness of safety margins that should be excised with tumor to avoid local recurrence, while some researchers considered the thickness of resection margin is irrelevant with disease progression [7, 8]. Nevertheless, the existence of tumor PC is not a standard parameter for pathological analysis so far.

In the present study, we analyzed 560 patients with sRCC (no cystic RCC included) and identified three major sRCC morphological growth patterns based on the growth types and features of peritumoral PC, indicating biological and oncological differences: single nodular pattern (SNP), multinodular fusion pattern (MFP), and infiltration pattern (IP).

The objective of this retrospective study was to evaluate the clinicopathologic value of different histological growth patterns, characterize peritumoral PC in sRCC, and define the optimal resection margin of healthy parenchyma individually, for avoiding the risk of positive SM.

## Subjects and methods

### Subjects

In this multi-center retrospective study, a total of 560 consecutive patients (416 males and 144 females) diagnosed with sRCC and underwent kidney surgeries by laparoscopy from May 2010 to October 2017 (patients with hereditary RCC were not included) were included, with the median age of 63.2 **±** 11.1 years (17–85), as shown in Table 1. The major clinical symptoms included gross hematuria (37 patients), microscopic hematuria (96), and renal area pain (55), and the other 338 cases showed no obvious symptoms (not shown in the table). All patients included in the study received preoperative computed tomography (CT) or magnetic resonance imaging (MRI) of the abdomen, a chest x-ray, and ultrasonography of the urinary system. Four patients were identified with multiple lesions in the lung and diagnosed as RCC lung metastasis. The mean tumor diameter was 3.0 **±** 0.6 cm (0.5–4.0 cm), 46 cases were < 2.0 cm, 123 were 2.0–3.0 cm, and 391 were 3.1–4.0 cm, respectively. Overall, 149 (26.6%) cases received NSS and the other 411 (73.4%) received RN. The decision to proceed NSS or RN depended on the patients’ preoperative imaging results, medical history, age, patient preference, and physician counseling.

**Table 1:** Clinicopathologic characteristics

Characteristic	Value
No. of patients	560
***Age (years)***	
Range	17–85
Median	63.2
***Sex***	
Male	416 (74.3%)
Female	144 (25.7%)
***sRCC subtype***	
Clear cell	480 (85.7%)
Chromofobe	38 (6.8%)
Papillary	26 (4.6%)
Others	16 (2.9%)
***Growth pattern***	
SNP	438 (78.2%)
MFP	58 (10.4%)
IP	64 (11.4%)
***pT stage***	
1a	541 (96.6%)
3a	19 (3.4%)
***Fuhrman grade***	
1	137 (24.5%)
2	370 (66.1%)
3	42 (7.5%)
4	11 (1.9%)
***PC***	
Absent	48 (7.5%)
Present	512 (92.5%)

*sRCC* Small renal cell carcinoma, *SNP* A single nodular pattern, *MFP* A multinodular fusion pattern, *IP* An infiltration pattern, *pT* Pathologic tumor, *PC* Pseudocapsule

### Histological assessment

The tumor size, integrity of peritumoral PC, and infiltration status of renal parenchyma were observed by morphological examination and then fixed in a 10% formalin solution. The growth pattern of the tumor was assessed on archival 4 μm HE-stained tissue sections cut from formalin-fixed paraffin-embedded specimens from the tumor-kidney boundary. In order to standardize the materials, only the tissue section with the highest representation of the interface was examined. All specimens were analyzed separately by two dedicated uropathologists and cases in doubt were judged through the consensus review.

### Statistical analysis

The *χ*^2^ test and Fisher’s exact probability test were utilized to compare the relationship between growth patterns and clinical or pathological variables. A *p* value < 0.05 was considered statistically significant. The statistical calculations were performed using SPSS version 22.0 (SPSS, Chicago, IL). For survival statistics, the Kaplan-Meier method was used to estimate the 5-year OS and CSS, and the log-rank test was conducted to compare the groups of patients with respect to the pT stage, growth patterns, and PC invasion. The survival curves were plotted with Graphpad Prism 8.0.1.

## Results

### Characteristics of tumor growth patterns

**Fig. 1:**
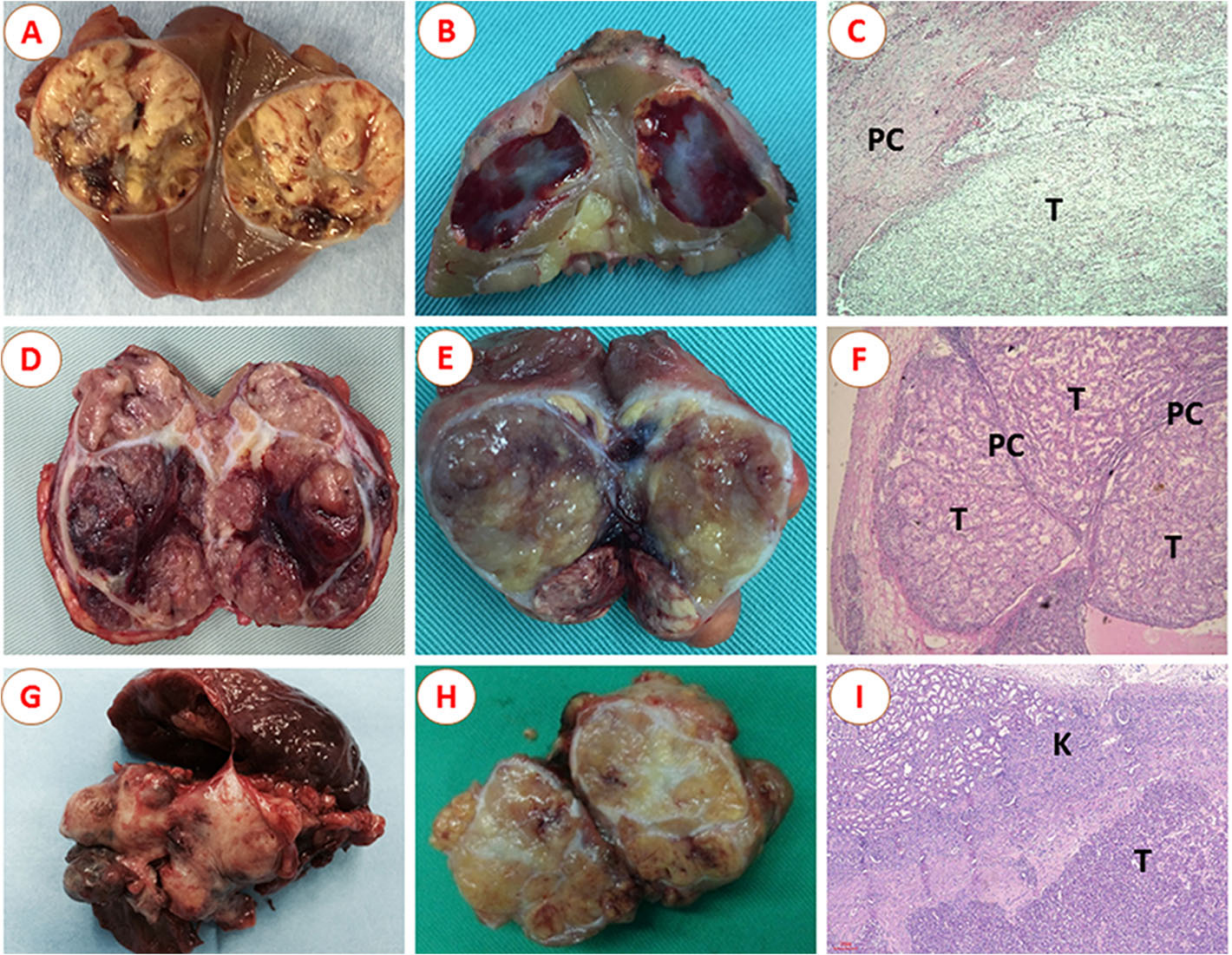
Different morphological subtypes of small renal cell carcinoma (sRCC) after nephron-sparing surgery: **A**, **B** Single nodular growth pattern of sRCC and the H-E staining pathological specimen (**C**); **D**, **E** Multinodular fusion growth pattern of sRCC and H-E staining pathological specimen (**F**); **G**, **H** Infiltration growth pattern of sRCC and H-E staining pathological specimen (**I**). Abbreviations: T, tumor; PC, pseudocapsule; K, kidney

The 560 renal tumor specimens were classified into three different growth patterns, with the examples shown in Fig. 1. We defined the three growth patterns of sRCC as follows: (I) SNP, only one entire tumor lesion exists in the kidney and the margin between tumor and renal parenchyma is clearly visible, and intact peritumoral PC could be observed in most of this type of sRCC (Fig. 1A–C); (II) MFP, several masses fuse into a large, well-defined, irregularly shaped mass, which usually separate from each other with connective fibrous tissues, and most of MFP tumors have complete peritumoral PC (Fig. 1D–F); and (III) IP, the tumor involves with poorly circumscribed margins with cancer cells extensively infiltrating and unequivocally entrapping normal kidney parenchyma, or the presence of normal renal tissue in the tumor, regardless of tumor circumscription (Fig. 1G–I). The peritumoral PC was defined as a band of fibrous connective tissues located at the interface between the tumor and adjacent parenchyma or adjacent tumors. Positive SM was defined as tumor reaching the edge of the specimen that was removed in the case of invasive tumor within 1 mm of the edge of the specimen. The tumor size, pathological subtype, tumor stage, Fuhrman grade, depth of tumor invasion, and other variables were also assessed entirely.

### Classification of PC

**Fig. 2:**
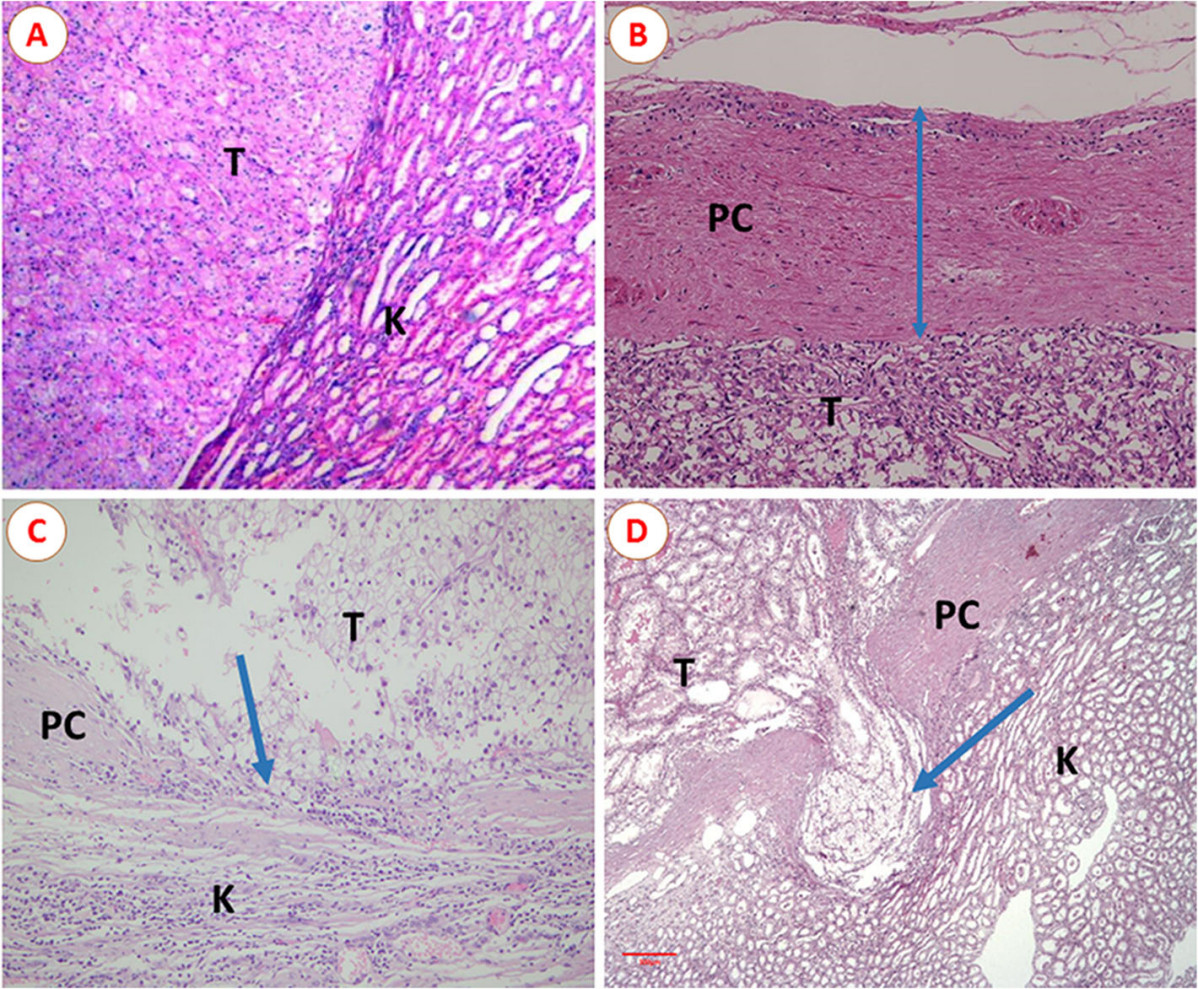
**A** No pseudocapsule between tumor and renal parenchyma. **B** Pseudocapsule (PC) intact and without infiltration of tumor cells (invasion (−)). **C** PC infiltrated by tumor cells but with no invasion beyond it (invasion (+)). **D** PC with neoplastic infiltration beyond it (invasion (++)), with the depth of tumor invasion at about 14 mm (the scale bar: 300 μm). Abbreviations: T, tumor; K, kidney

The specimen with no obvious peritumoral PC was shown in Fig. 2A. PC status could be further divided into three categories: PC intact and free from invasion (invasion (−), Fig. 2B), PC with neoplastic infiltration on the parenchymal kidney with no invasion beyond it (invasion (+), Fig. 2C), and PC with neoplastic infiltration and invasion beyond it (invasion (++), Fig. 2D).

### Baseline clinicaopathologic characteristics

The descriptive clinicopathologic statistics for this study are provided in Table 1. A total of 560 patients were included, with the median age of 63.2 (17 to 85), in which 416 patients were males and the others were females. The histopathologic evaluation based on the 2004 WHO classification revealed that 480 (85.7%) cases were clear cell RCC, 38 (6.8%) were chromophobe RCC, 26 (4.6%) were papillary RCC, and 16 (2.9%) were other RCC subtypes. The SNP of tumors was found in 78.2% patients (*n* = 438), MFP in 10.4% (*n* = 58), and IP in 11.4% (*n* = 64) of all patients.

The post-operative pathological analysis based on the TNM classification showed that 96.6% (*n* = 541) of tumors were pT1a, and 3.4% (*n* = 19) were at the pT3a stage, as the perirenal adipose was infiltrated by tumor cells. According to the Fuhrman nuclear grading, 24.5% (*n* = 137) of the tumors were Grade I, 66.1% (*n* = 370) were Grade II, 7.5% (*n* = 42) were Grade III, and 1.9% (*n* = 11) were Grade IV. The presence of peritumoral PC was detected in 512 (92.5%) cases, and the rest of 48 (7.5%) patients showed no obvious PC.

### Distribution of growth patterns in sRCC subtypes

Table 2 showed the distribution of three growth patterns in different sRCC subtypes, and no statistical difference was found in such a situation (*p* = 0.941). According to the results, the distribution of three growth patterns was SNP (69.2–81.3%) > MFP(6.2–15.4%) > IP(10.5–15.4%) in all sRCC subtypes.

**Table 2:** The distribution of three growth patterns in different sRCC subtypes

sRCC subtype	Growth patterns			***p*** value
	SNP	MFP	IP	
Clear cell	378 (78.8%)	48 (10.0%)	54 (11.2%)	0.922
Chromofobe	29 (76.3%)	5 (13.2%)	4 (10.5%)	
Papillary	18 (69.2%)	4 (15.4%)	4 (15.4%)	
Others	13 (81.3%)	1 (6.2%)	2 (12.5%)	

*sRCC* Small renal cell carcinoma, *SNP* A single nodular pattern, *MFP* A multinodular fusion pattern, *IP* An infiltration pattern

### Relationship of PC, PC invasion, pT stage, SM, and different growth patterns

The results suggested that the presence of PC was significantly correlated with different morphological growth patterns (*p* < 0.001), and the absence of PC in IP (43.8%) was obviously more frequent than the other two patterns (Table 3). PC status was also statistically associated with different growth patterns (*p* < 0.001) (Table 3), the situation of invasion (++) in IP was significantly higher than SNP (*p* < 0.001) and MFP (*p* < 0.001) (not shown in the table). In all specimens of invasion (++), the mean depth of tumor invasion in renal parenchyma was 1.06 mm (SD: 0.22; median: 1.11; range: 0.30–2.6 mm), the infiltrative depth in more than 95% cases was limited in 2 mm and 100% in 3 mm beyond the surface of peritimoral PC.

**Table 3:** The Relationship of PC, PC Invasion, pT stage, SM, and different growth patterns

Growth pattern		SNP	MFP	IP
**PC**	**Absent**	17 (3.9%)	3(5.2%)	28 (43.8%)
	**Present**	421 (96.1%)	55(94.8%)	36 (56.2%)
***p*value**		< 0.001		
**PC invasion**	**Invasion (−)**	325 (77.2%)	35(63.6%)	0 (0%)
	**Invasion (+)**	90 (21.4%)	16(29.1%)	8 (22.2%)
	**Invasion (++)**	6 (1.4%)	4(7.3%)	28 (77.8%)
***p*value**		< 0.001		
**pT stage**	**T1a**	436 (99.5%)	55(94.8%)	50 (78.1%)
	**T3a**	2 (0.5%)	3(5.2%)	14 (21.9%)
***p*value**		< 0.001		
**SM**	**(+)**	0 (0%)	1(3.8%)	2 (5.9%)
	**(−)**	89 (100%)	25(96.2%)	32 (94.1%)
***p*value**		0.088		

*PC* Pseudocapsule, *SNP* A single nodular pattern, *MFP* A multinodular fusion pattern, *IP* An infiltration pattern; Invasion (−), PC intact and free from invasion; Invasion (+), PC with neoplastic infiltration on the parenchymal kidney with no invasion beyond it; Invasion (++), PC with neoplastic infiltration and invasion beyond it. *pT* Pathologic tumor, *SM*, Surgical margins

A total of 19 patients were diagnosed with pT3a through post-operative pathological examination, in which 14 were found in IP cases. Table 3 showed that pT3a stage was statistically related to IP (*p* < 0.001), and the ratio of such stage in IP was significantly higher than SNP (*p* < 0.001) and MFP (*p* = 0.007). In our cohort, a total of 149 sRCC patients underwent NSS eventually, and 3 (2.0%) of them showed positive SM in the following pathological findings (Table 3), 1 case in MFP and 2 cases in IP. No significant differences of SM (+) were discovered between SNP and MFP (*p* = 0.226) or between SNP and IP (*p* = 0.075). The situation of SM did not seem to vary across the three growth patterns (*p* = 0.088), while these data were only available for 149 cases, and only 3 patients were SM (+).

### Survival outcomes

**Fig. 3:**
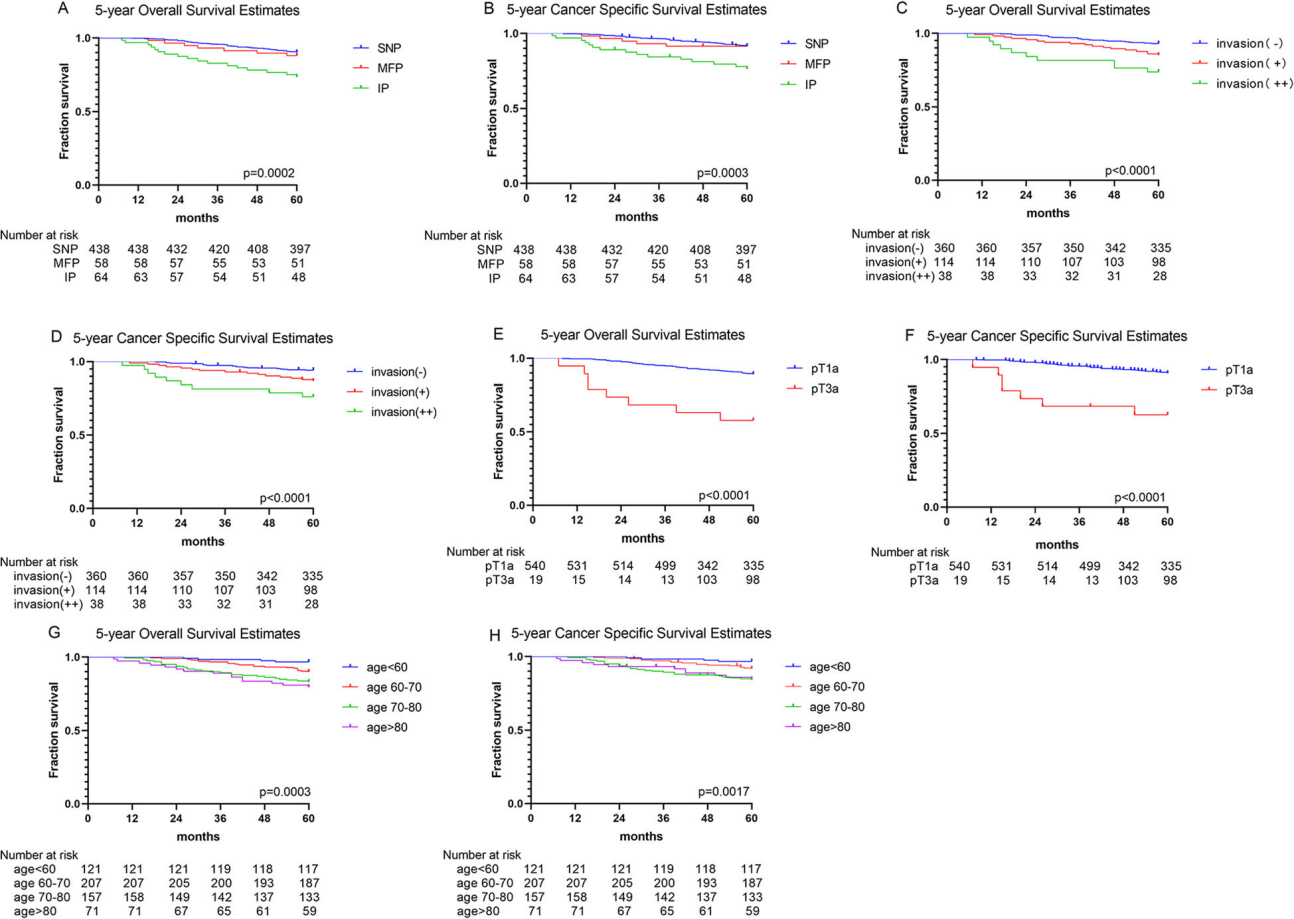
Five-year OS and CSS among different morphological growth patterns (**A**), PC status (**B**), pT stages (**C**), and age (**G**, **H**). Abbreviations: OS, overall survival; CSS, cancer-specific survival; PC, pseudocapsule

The median follow-up time was 62.7 months, and there were 64 deaths and 57 cancer-specific deaths. Among all the cancer-specific deaths, 3 (5.3%) deaths were caused by local recurrence, and others were caused by metastasis. Thirty-one (54.4%), 5 (8.8%), 2 (3.5%), and 1(1.8%) deaths were caused by metastasis to single organ, including lung, bone, liver, and brain, respectively. Fifteen (26.3%) deaths were caused by distant metastasis where more than one organ. We calculated and compared the 5-year OS and CSS in different morphological growth patterns, PC status, and pT stage. For morphological growth patterns, the 5-year OS were 90.2%, 87.9%, and 73.4% in SNP, MFP, and IP (Fig. 3A), and the 5-year CSS were 91.5%, 91.4%, and 76.2%, respectively (Fig. 3B). There were statistical differences of the 5-year OS (*p* = 0.0002) and the 5-year CSS (*p* = 0.0003) between the three different growth patterns. The 5-year OS (*p* < 0.0001) and CSS (*p* < 0.0001) in SNP were significantly higher than that in IP (not shown in the table). For PC status, the 5-year OS were 93.1%, 85.1%, and 73.7% in invasion (−), invasion (+), and invasion (++) (Fig. 3C), and the 5-year CSS were 94.1%, 86.8%, and 76.2%, respectively (Fig. 3D). There were statistical differences of the 5-year OS (*p* < 0.0001) and the 5-year CSS (*p* < 0.0001) between PC status. The 5-year OS and CSS in invasion (++) and invasion (+) were significantly higher than that in invasion (−) (not shown in the table). For the pT stage, the 5-year OS were 89.1% and 57.9% in pT1a and PT3a (Fig. 3E), and the 5-year CSS were 90.7% and 62.7%, respectively (Fig. 3F). There were statistical differences of them between the pT1a and pT3a stage. For stratified analysis, older people had poor 5-year OS (*p* = 0.0003) and CSS (*p* = 0.0017) with statistical differences than young (Fig. 3G, H).

## Discussion

At present, sRCC (≤ 4 cm) is generally considered to be well differentiated, with low clinical stage and better prognosis. Several studies have shown that NSS could provide equally effective local control and oncologic safety as compared with RN for treating sRCC [9–11]. In addition, some reports have also revealed that NSS could offer local tumor control equivalent to RN, even for RCC of more than 4 cm [12–14]. One meta-analysis indicated that, as compared with RN, NSS reduced the incidence of post-operative renal complication by 61%, and the mortality of patients by 19% [15]. These advantages make NSS become the primary treatment for sRCC currently. Nevertheless, some sRCC masses with an infiltrative growth pattern show an aggressive clinical course and high tendency for distant metastasis. It is therefore extremely important to systematically study the histological characteristics of sRCC and to investigate the appropriate operational mode.

Akitoshi et al. reported that the growth pattern could be a predictive parameter for small clear cell RCC [16]; moreover, in their subsequent study, they demonstrated that the infiltrative growth pattern was an independent risk factor for disease-free survival (DFS) and CSS even in advanced cases [3]. In our study, we divided the growth patterns of sRCC into three groups: SNP, MFP, and IP, and we found most tumors (78.2%) consist of a single mass. Peritumoral PC is mainly composed of connective fibrous tissue with the mean thickness of 0.39 mm (SD: 0.33; median: 0.58; range: 0.2–1.0 mm). Some researchers supposed that it was a kind of protective manner to confine tumor growth and proliferation, because inflammatory layer consisted of lymphocytes, and plasmocytes sometimes could also be found between PC and normal parenchyma [17]. Minervini et al. analyzed 90 cases of pT1 stage RCC tumors which underwent enucleation and found complete PC was present in 67% of tumors, and incomplete PC or PC infiltrated by cancer cells was detected in the rest of the samples [18]. Our study showed that 360 (64.3%) cases had intact PC, and the total proportion of PC invaded by tumor cells in IP (100%) was remarkably higher than that of the other two growth patterns. Accordingly, tumor enucleation is not recommended in sRCC of IP owing to the high risk of incomplete excision, so extending the scope of resection around the tumor is highly essential.

To prevent the risk of local recurrence, the excision of a minimal and visible margin of normal-appearing parenchyma around the tumor is regarded as the optimal surgical technique of NSS. However, whether or not to excise a rim of healthy parenchyma, theoretically adequate to avoid a positive SM and local recurrence, is still controversial. Marco et al. reviewed literatures published from January 2002 to May 2012 and discovered the overall incidence of positive SM ranges from 0 to 7%, with no significant differences between the open, laparoscopic, and robot-assisted techniques [19, 20]. A very similar result was obtained in our study, with the incidence of positive SM of 2.0%. Although no statistical differences of positive SM were discovered across the three growth patterns, the probability of positive SM in MFP and IP was evidently higher than that in SNP, as revealed in our study. The pathological stage of 19 (3.4%) patients who were diagnosed pT1a stage before operation increased to the pT3a stage after surgery as the perirenal adipose tissue was invaded with tumor cells, suggesting that NSS might lead to positive SM of sRCC, though most neoplasms were limited in PC. Seongyub Oh reported that perirenal fat infiltration was an independent prognostic factor for predicting DFS in patients with tumors of 7 cm or less in size, and the presence of perirenal fat infiltration required stricter follow-up planning, even in small renal cell carcinoma [21]. Considering this, it is necessary to routinely excise the perirenal adipose tissue to reduce the possibility of local recurrence when undergoing NSS.

Therefore, the surgical margin (SM) is recommended to proceed along with the peritumoral PC for patients with SNP sRCC, because most SNP tumors were surrounded by complete PC. Tumor enucleation is also an available treatment for this kind of patient. 63.6% of MFP tumors have intact peritumoral PC, even if they are unlikely to grow in an irregular or polymorphy manner. For this pattern, tumor enucleation ought to be particularly choosy and a NSS margin with the thickness of 1–3 mm of renal parenchyma around the mass should be acceptable. Tumor enucleation is not suitable in IP sRCC in which almost all tumors possess no PC or incomplete PC infiltrated with cancer cells. Since the infiltrative depth of all tumors was confined in the range of 3 mm away from the peritumoral PC, as shown in the study, it is recommended to extend the optimal resection distance of NSS in IP sRCC more than 3 mm around the tumor surface, to acquire a histologic tumor-free margin. As for some infrequent cases, e.g., tumor cells invade deeply in normal kidney parenchyma over PC and sRCC with the presence of tumor multifocality or satellite lesions [22, 23], the recommended surgical measure of NSS is not appropriate any more, and radical nephrectomy should be considered [24, 25]. Our study also proved that prognostic outcome of SNP and MFP was significantly better than that in IP, and the infiltration level of PC invasion was closely related to the 5-year OS and CSS.

There are some limitations of our study. First and uppermost, no reliable method is available to precisely distinguish different growth patterns before the operation at present, which confines the application of growth patterns in clinics. Second, the study was a retrospective, nonrandomized design which decreased the level of evidences. Third, pathologic data were collected from multiple centers over a long period, and the procedures for handling specimens were not uniform. Last, there is an inherent bias for the quality of specimens operated with different surgical manners that can affect the SM. Nevertheless, we believe that recognition of different growth patterns is useful for preoperative decision-making in the future. Large-scale studies are warranted to validate our growth pattern classification, to determine the optimal resection range.

## Conclusions

According to our study, the growth pattern of sRCC could be divided into three morphological subtypes: SNP, MFP, and IP, and the results showed that incomplete peritumoral PC, pT stage, and positive SM were statistically correlated with IP. As shown in the study, morphological growth patterns, if validated externally in a larger cohort, could be used as a valuable biomarker to help operating NSS in sRCC patients to avoid the risk of positive SM. The effectiveness of the growth pattern as a novel proposed parameter to direct NSS in sRCC patients deserves further exploration.

## Data Availability

The datasets used and analyzed during the current study are available from the corresponding author on reasonable request.

## Abbreviations

sRCC: Small renal cell carcinoma
NSS: Nephron-sparing surgery
RN: Radical nephrectomy
PC: Pseudocapsule
SM: Surgical margin
RCC: Renal cell carcinoma;
SNP: Single nodular pattern
MFP: Multinodular fusion pattern
IP: Infiltration pattern
OS: Overall survival
CSS: Cancer-specific survival
DFS: Disease-free survival
